# The systemic lupus erythematous in paediatrics: Moroccan experience of a unit of pediatric rheumatology

**DOI:** 10.1186/1546-0096-12-S1-P328

**Published:** 2014-09-17

**Authors:** Kenza Bouayed, K Bouayed, N Echcharaï, N Mikou

**Affiliations:** 1 CHU Ibn Rochd, Casablanca, Morocco

## Introduction

Systemic Lupus Erythematous (SLE) is a chronic autoimmune disease characterized by multi-organ involvement. Its pathogenesis remains controversial. Diagnosis in children is also based on the ARA criteria (American Rheumatism Association).

## Objectives

We propose to report the epidemiological, clinical, immunological, therapeutic and evolutive profile of childhood’s SLE through this retrospective study in the Pediatric Rheumatology Service of the Children's Hospital of Casablanca, conducted between December 2001 and January 2014.

## Methods

We report a series of 30 children, including 25 girls, mean age of onset is 12 years, with a range of 7-16 years, the average time from diagnosis is 6 months. The mode of presentation of the disease is classical (general signs, skin and joints involvement) in 56% of cases, by lupus nephritis in 26.66 % of patients, or a Macrophage Activation Syndrome “MAS” in 13.33 % of cases and lupus nephritis associated with MAS in one patient.

## Results

The clinical picture showed fever in 76.66% of patients, the frequency of joint locations (90%), skin (86.66%), kidney (66.6%) , hematologic (50%) lung (36.66%), gastrointestinal (30%), neuropsychiatric (26.66%) and cardiac (23.3%) involvement.

The hematological involvement was detected in 76.66% of our patients, an inflammatory syndrome in 83.33%, immunological disturbances with positive titers of anti- DNAN AC (90%) , ANA (93.6%), and a reduction of the complement (83.33%) . False syphilis serology completed by anti β2Glycoprotein1 antibodies (26%) and anti- cardiolopine were positive in respectively 40, 26 and 20% of cases. Renal involvement is manifested by renal insufficiency in 43% of cases, a significant proteinuria in 50% of cases and prevalence of class IV on biopsy. A case of kikushi fujimoto has been reported as 5 hemophagocytic syndromes.

All patients were treated with systemic corticosteroids and hydroxychloroquine. The use of methylprednisolone bolus was indicated in cases of SAM or severe renal impairment associated with cyclophosphamide / MMF and anti-proteinuric medication.

## Conclusion

The prognosis of pediatric SLE remains unpredictable; however it is attached to the renal, neurological and the occurrence of MAS. We mourn two deaths one by neurological attack and other by renal failure.

## Disclosure of interest

None declared.

